# Anti-cancer effect and gene modulation of ET-743 in human biliary tract carcinoma preclinical models

**DOI:** 10.1186/1471-2407-14-918

**Published:** 2014-12-05

**Authors:** Caterina Peraldo-Neia, Giuliana Cavalloni, Marco Soster, Loretta Gammaitoni, Serena Marchiò, Francesco Sassi, Livio Trusolino, Andrea Bertotti, Enzo Medico, Lorenzo Capussotti, Massimo Aglietta, Francesco Leone

**Affiliations:** University of Turin Medical School, Department of Oncology, IRCCS-Candiolo, Strada provinciale 142, Km 3.95, Candiolo, 10060 Turin, Italy; Fondazione del Piemonte per l’Oncologia (FPO), IRCCS-Candiolo, Strada provinciale 142, Km 3.95, Candiolo, 10060 Turin, Italy; Laboratory of Tumor Microenvironment, IRCCS-Candiolo, Strada provinciale 142, Km 3.95, Candiolo, 10060 Turin, Italy; University of Turin Medical School, Unit of Molecular Pharmacology, IRCCS-Candiolo, Strada provinciale 142, Km 3.95, Candiolo, 10060 Turin, Italy; Laboratory of Oncogenomics, IRCCS-Candiolo, Italy, Strada provinciale 142, Km 3.95, Candiolo, 10060 Turin, Italy; Department of General Surgery and Surgical Oncology, Ospedale Mauriziano Umberto I, Strada provinciale 142, Km 3.95, Candiolo, 10060 Turin, Italy

**Keywords:** Biliary tract carcinoma, ET-743, Preclinical model, Chemotherapy, Patient-derived xenograft

## Abstract

**Background:**

Standard chemotherapy in unresectable biliary tract carcinoma (BTC) patients is based on gemcitabine combined with platinum derivatives. However, primary or acquired resistance is inevitable and no second-line chemotherapy is demonstrated to be effective. Thus, there is an urgent need to identify new alternative (chemo)therapy approaches.

**Methods:**

We evaluated the mechanism of action of ET-743 in preclinical models of BTC. Six BTC cell lines (TFK-1, EGI-1, TGBC1, WITT, KMCH, HuH28), two primary cell cultures derived from BTC patients, the EGI-1 and a new established BTC patient-derived xenografts, were used as preclinical models to investigate the anti-tumor activity of ET-743 *in vitro* and *in vivo*. Gene expression profiling was also analyzed upon ET-743 treatment in *in vivo* models.

**Results:**

We found that ET-743 inhibited cell growth of BTC cell lines and primary cultures (IC50 ranging from 0.37 to 3.08 nM) preferentially inducing apoptosis and activation of the complex DNA damage-repair proteins (p-ATM, p-p53 and p-Histone H2A.x) *in vitro*. In EGI-1 and patient-derived xenografts, ET-743 induced tumor growth delay and reduction of vasculogenesis. *In vivo* ET-743 induced a deregulation of genes involved in cell adhesion, stress-related response, and in pathways involved in cholangiocarcinogenesis, such as the IL-6, Sonic Hedgehog and Wnt signaling pathways.

**Conclusions:**

These results suggest that ET-743 could represent an alternative chemotherapy for BTC treatment and encourage the development of clinical trials in BTC patients resistant to standard chemotherapy.

**Electronic supplementary material:**

The online version of this article (doi:10.1186/1471-2407-14-918) contains supplementary material, which is available to authorized users.

## Background

Biliary tract carcinoma (BTC) is a particularly lethal malignancy arising from the ductal epithelium of the biliary tree, either within the liver or from the extrahepatic bile ducts [1]. Most patients with BTC are diagnosed at advanced stages, and have a life expectancy of <12 months [2].

Chemotherapy is commonly used to improve patients’ outcome and to control tumor progression. Different chemotherapeutic agents have been employed [3]; few randomized trials have established the combination of gemcitabine (GEM) and platinum compounds to be the standard of therapy for unresectable BTC patients [4–6]. In these studies, overall survival (OS) in the GEM and platinum combination arm was of about 11 months. These studies, therefore, demonstrated that there are very limited possibilities for prolonging survival of BTC patients, and that it is crucial to find novel therapeutic strategies for the treatment of BTC patients.

Ecteinascidin-743 (ET-743), a compound isolated from the marine tunicate *Ecteinascidia turbinata*[7, 8] with a potent cytotoxic activity against a variety of tumors *in vitro* and *in vivo*[9, 10], has been approved for treatment of soft-tissue sarcoma and ovarian cancer [11, 12]. Its mechanism of action is linked to binding to the minor groove of DNA and to a variety of modulatory effects on the tumor microenvironment, including changes in the production of several inflammatory mediators like the chemokines CCL2 and CXCL8, the cytokine IL-6 and the angiogenic factor VEGF [13]. Chronic inflammation contributes to cancerogenesis and disease progression in different types of solid tumors [14, 15]. Tumor-associated macrophages (TAMs) represent the major class of immune cells within the tumor microenvironment [16] and have been shown to promote tumor proliferation, increase invasiveness and mitigate T cell-mediated cytotoxic antitumor responses [17–19]. They are regarded as potential targets in anticancer therapies and, in this context, ET-743 may represent a suitable tool to overcome myelomonocytic cell-mediated exacerbation of the malignant phenotype and immune suppression [20]. In BTC the presence of TAM has been documented and correlated with a worse prognosis [21] but there are no preclinical data of ET-743 activity in BTC. Literature reports only the anecdotal case of a BTC patient involved in a phase I study who experienced a complete metabolic response with ET-743 [22].

Here, we investigated the potential anti-tumor activity of ET-743 and its effect on gene expression profiling in human preclinical models of BTC.

## Methods

### Cell lines and patients

The extrahepatic cholangiocarcinoma (ECC) cell lines TFK-1 and EGI-1, the intrahepatic cholangiocarcinoma (ICC) cell line HuH28 and the gallbladder carcinoma (GBC) cell line TGBC1 (Cell Bank, RIKEN Bioresource Center Riken Cell Bank, Japan) were cultured in RPMI 1640 containing 10% fetal bovine serum (FBS) (all from Sigma–Aldrich, St. Louis, MO, USA), 100 U/mL penicillin and 100 μg/mL streptomycin (P/S) (Life Technologies Gathersburg, MD). The ECC WITT cells and the ICC mixed to hepatocarcinoma KMCH cells (provided by Dr. Andersen, Laboratory of Experimental Carcinogenesis, National Institutes of Health, Bethesda, Maryland), were cultured in DMEM (Sigma–Aldrich) plus 10% FBS. The authentication of all the cell lines was performed by using Cell_ID system (Promega, Corporation, Madison, WI, USA) comparing their profiles with those published on the DMSZ database. Human endothelial cells Huvec were cultured on gelatin-coated plastic, in Medium 199 plus 20% FBS, added of P/S, 50 μg/ml of heparin (Sigma–Aldrich,) and 100 μg/ml of bovine brain extract (Sigma–Aldrich).

Primary cell cultures were isolated from peritoneal liquid obtained by paracentesis procedure from two patients with ICC. They received the combination of gemcitabine and oxaliplatin and progressed after 5 and 2 months, respectively. Only CK7/19 positive cells were cultured in KODMEM/F12 (Life Technologies) plus 10% FBS.

Biological material for the set-up of primary cell cultures and patient derived xenograft (PDX or xenopatient) protocol were obtained from ICC patients who have signed the informed consent, following institutional review board-approved protocols “PROFILING Protocol, n° 001-IRCC-00 IIS-10” approved by Comitato Etico Interaziendale of A.O.U. San Luigi Gonzaga, Orbassano, Torino, Italy). This institutional study provides molecular genetic analysis, set up of primary cultures and the creation of PDX from tumor biological samples (primary tumor, metastasis, tumor cells taken under paracentesis or thoracentesis procedures, and blood) from patients with colorectal cancer, prostate cancer, head and neck cancer, primary tumors of the stomach, primary tumors of the liver and biliary tract, glioblastoma, ovarian or primary tumors with metastases, primary tumors of the lung, primary tumors of the breast, rare cancers defined by incidence ≤ 5×10^6^, sarcomas and metastatic melanomas.

### Drugs

ET-743 from PharmaMar (Pharma Mar, S.A., Madrid, Spain) was dissolved in PBS at 1.3 mM for *in vivo* experiments, and at a concentration of 1 μM in dimethyl sulfoxide (DMSO) (Sigma-Aldrich) for *in vitro* experiments, and stored at -20°C. For *in vitro* experiments, 0.001% DMSO was added to ET-743-untreated cells.

### Cell growth assay

Cells (3000/well) were seeded onto 96-well tissue culture plates; after 24 hours they were treated with escalating doses of ET-743 (0.078-10 nM) in appropriate culture medium added of 10% FBS for another 72 hours. Cell growth was evaluated with the Cell Titer-Glo® cell viability assay (Promega). All tests were performed in quadruplicate and repeated in three independent experiments. IC50 values, dose of drug that inhibits 50% of the cell growth compared with control calculated for each cell line after 72 hours of drug treatment, was calculated using the CalcuSyn software, based on the Chou-Talalay method.

### DNA content and apoptosis analysis

To determine the cell cycle status, 1×10^6^ cells/well were seeded onto six-well tissue culture plates for 24 hours, and then treated with 5 nM ET-743 for an additional 48 hours. Cells were then fixed in 70% ethanol at -20°C for 16 hours followed by washing in PBS, resuspended in staining solution (50 μg/ml Propidium Iodide, PI +100 μg/ml RNaseA in PBS) (Sigma–Aldrich) and overnight incubated at 4°C. For apoptosis analysis, 1 × 10^5^ cells were washed in Binding Buffer (0.01 M Hepes, 0.15 M NaCl, 5 mM CaCl_2_), stained with allophycocyanin (APC) conjugated AnnexinV/PI (Bender MedSystems Wein, Austria) and incubated for 15 minutes at room temperature. DNA content and apoptosis level were determined by flow cytometry using Cyan and Summit Research Software (Dako, Glostrup, Denmark). Three independent experiments were performed.

### Western blot

Cells were lysed in boiling buffer (10% SDS, 0.5 M Tris–HCl pH 6.8) and centrifuged at 20,000×*g* for 30 minutes; 20 μg of protein were separated on 7.5-15% SDS-PAGE and transferred onto 0.45-μm nitrocellulose membranes (GE Healthcare Europe, Milan, Italy). Blots were stained using standard procedures and signals were revealed by a chemiluminescence reagent (Euroclone, Milan, Italy). Horseradish peroxidase (HRP)-linked secondary antibodies, anti-phospho-Ataxia Telengectasia (Ser 1981) (ATM), anti-ATM, anti-phospho-p53, anti-p53 and anti-vinculin were from Cell Signaling Technology (Beverly, USA); anti-phospho-Histone H2A.x (Ser 139) was from Millipore (Temecula, CA) and anti-Histone H2A.x was from GeneTex (San Antonio, Texas). Three independent experiments were performed.

### Antitumor activity of ET-743 in *in vivo*models of BTC

We investigated the antitumor activity of ET-743 in preclinical *in vivo* models of BTC. For *in vivo* studies, NOD (Non-Obese Diabetic)/Shi-SCID (severe combined immunodeficient) female mice (4–6 weeks old) (Charles River Laboratory) were maintained under sterile conditions in micro-isolator cages at the animal facilities of the IRCCS-Candiolo. All animal procedures were approved by the Institutional Ethical Committee for Animal Experimentation (Fondazione Piemontese per la Ricerca sul Cancro) and by the Italian Ministry of Health.

In three independent experiments, 14 mice, either in EGI-1-xenograft (5×10^6^ cells/mouse subcutaneously injected), or an ICC patient derived xenograft model [23], here named CHC001PDX, were randomized to receive intra venous (i.v.) weekly 0.15 mg/Kg ET-743 [24–26] or drug vehicle (PBS) for 3 weeks (7 mice for arm of treatment). Tumor size was measured weekly. Volumes were calculated using the formula V = AxB^2^/2 (V = tumor volume, A = largest diameter; B = smallest diameter). Mean volumes of treated and untreated xenografts were compared by two-way Anova, considering a p-value <0.05 (C.I. 95%) as statistically significant. Tumors were formalin-fixed, paraffin-embedded (FFPE) for immunohistochemical evaluations.

### Immunohistochemistry on BTC *in vivo*models

For the evaluation of ET-743 effects on tumor xenografts, tissue sections were stained with anti-Ki67/MIB1 (Dako) and anti-CD31 (BD) antibodies, followed by incubation with secondary antibody (Invitrogen). Ki67 expression was evaluated in 10 fields for each section at 40x by ImageJ; CD31 quantification was performed on 10 z-stack images for each slide at 20× magnification by calculating the positively stained vessel area. Expression values of treated and untreated xenografts were compared by two tailed unpaired *T*-test, assuming a p-value <0.05 (C.I. 95%) as statistically significant.

### Microarray analysis

For gene expression profiling (GEP), total RNA was extracted by using TissueLyser LT (Qiagen) and then purified by Absolutely RNA miRNA kit (Agilent Technologies), following manufacturers’ protocols. Quantitative and qualitative evaluation of total RNA was performed by Nanodrop and BioAnalyzer respectively. For GEP analysis, 100 ng of total RNA was amplified and labeled using Low Input Quick Amp Labeling Kit, one-color kit (Agilent Technologies). Six hundred ng of labeled RNA were hybridized on SurePrint G3 Human Gene Expression 8×60K v2 glass arrays. The experiment was carried out by two technical replicates. Arrays were scanned and images analyzed by the Feature Extraction Software from Agilent Technologies (version 10.7), and raw data were then processed using the Bioconductor package Limma (Linear models for microarray analysis). Background correction was performed with the *normexp* method with an offset of 50, and *quantile* was used for the between-array normalization. The empirical Bayes method was used to compute a moderated t-statistics [27]. The threshold for |log FC| of 0.58 and a *P* value <0.05 was used to identify modulated transcripts. Microarray data were deposited in Gene Expression Omnibus (GSE63043).

## Results

### ET-743 induces cell cycle perturbation, apoptosis, and activation of proteins involved in DNA damage-repair in biliary tract carcinoma cells in vitro

**Figure 1:**
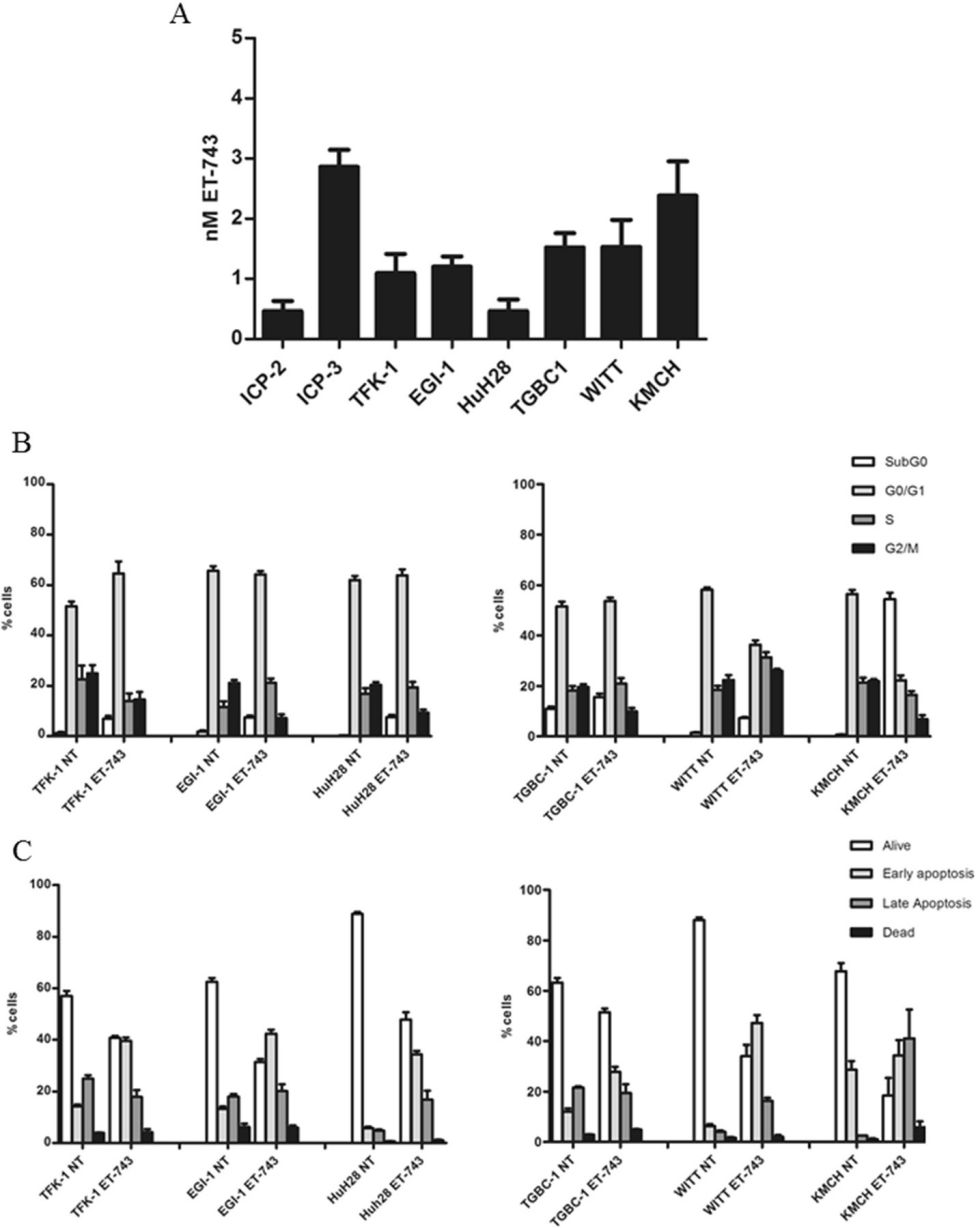
**Effect of ET-743 on proliferation, cell cycle and apoptosis** ***in vitro*** **. (A)** ET-743 inhibits growth *in vitro*. Cells were treated with escalating doses of ET-743 (0.078-10 nM) for 72 hours and cell growth was evaluated by Cell Titer-Glo® cell viability assay. IC50 values were calculated using the CalcuSyn software, based on the Chou-Talalay method. ICP-2/3: primary cells derived from two intrahepatic cholangiocarcinoma (ICC) patients. TFK-1, WITT and EGI-1: extrahepatic cholangiocarcinoma cell lines; HuH28 ICC cell line; TGBC1: gallbladder carcinoma cell line; KMCH: ICC mixed with hepatocarcinoma cell line. The histograms represent the mean of IC50 values (bars represent SEM) from three independent experiments. Effect of ET-743 on cell cycle **(B)** and on apoptosis **(C)** *in vitro*. BTC cell lines were treated with 5 nM of ET-743 for 48 h and subjected to cell cycle analysis and apoptosis detection by AnnexinV/PI staining by flow cytometry as described in methods. The bars represent the average with SEM of percentage of cells in each phase **(B)** and apoptotic cells **(C)** of three independent experiments. NT: no treated cells.

To investigate the capability of ET-743 to interfere with cell growth, BTC cell lines and primary cultures were treated with escalating doses (0.078-10 nM) of ET-743 for 72 hours. As indicated in Figure 1A, all the BTC cells proved to be sensitive to ET-743 treatment, with an IC50 ranging from 0.37 nM for ICP-2 cells to 3.08 nM for ICP-3 cells. Interestingly, the most responsive BTC cells, HuH28 and ICP-2, resulted resistant to gemcitabine (Additional file 1: Table S1) [28]. Cell cycle status changes and induction of apoptosis by ET-743 were also examined on BTC cells after 48 hours of treatment. As shown in Figure 1B and in Additional file 2: Figure S1, ET-743 caused a different distribution in cell cycle phases. The common evidence was an increment in the subG_0_ phase cell fraction, particularly evident in the KMCH cells, indicating that the growth inhibition by ET-743 could be mainly due to the induction of apoptotic cell death. A more specific assay, in fact, indicated that, after 48 hours of treatment, ET-743 induced apoptosis in the tested cell lines, as shown in Figure 1C and in Additional file 3: Figure S2. We investigated whether ET-743 was capable of activating the complex DNA damage-repair protein machine. For this purpose, BTC cells were treated for 24 hours with 5 nM ET-743 and analyzed for the expression level of p-ATM, p-p53, and p-Histone H2A.x. We observed that phosphorylation of ATM, Histone H2A.x, and p53 was increased upon ET-743 treatment (Additional file 4: Figure S3).

### ET-743 reduces tumor growth and angiogenesis in *in vivo*models of BTC

**Figure 2:**
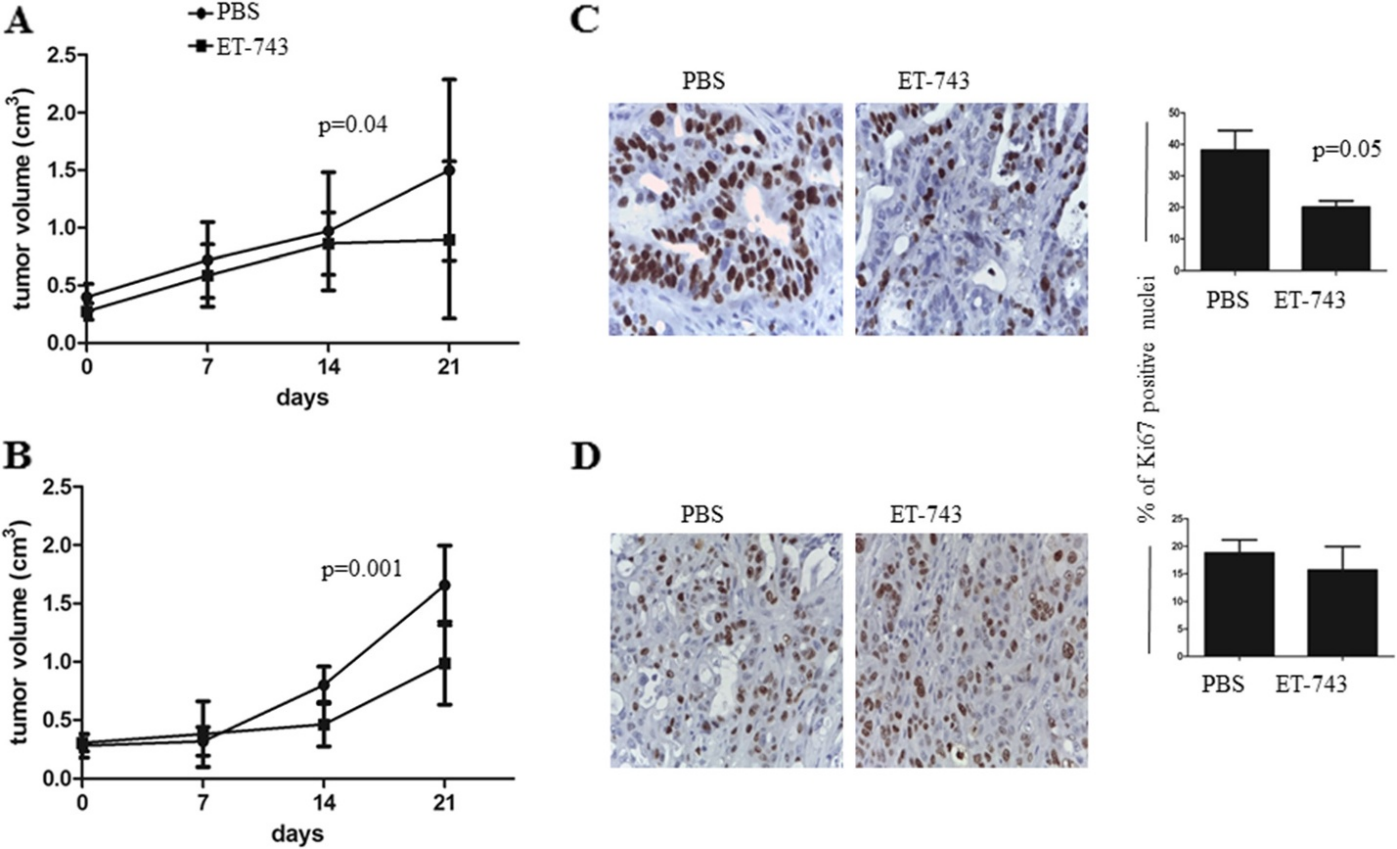
***In vivo*** **antitumor activity of ET-743 in human BTC preclinical models.** The graphs indicate the mean tumor volume (cm^3^) weekly measured: 0 (start of treatment), 7, 14, and 21 days after treatment with ET-743 (weekly 0.15 mg/Kg ET-743 or PBS in mice control cohort) (error bars: SEM). Seven mice for each arm of treatment in three independent experiments were used. One week after the last drug administration, a significant slow of tumor growth was shown in treated mice in both CHC001PDX **(A)** and EGI-1-xenografts **(B)**. Ki67 staining of section derived from CHC001PDX **(C)** and EGI-1 xenograft **(D)** tumors treated with PBS or ET-743 and relative quantification. A statistical significant reduction of Ki67 positive cells was revealed in the ET-743-treated CHC001PDX group.

The antitumor activity of ET-743 was investigated in BTC preclinical *in vivo* models, both on EGI-1 xenograft and in CHC001PDX. Each cohort of xenografts was randomized to receive ET-743 (0.15 mg/Kg) or drug vehicle (PBS) i.v. weekly for 3 weeks following the schedule of treatment of D’Incalci and coll. in other human xenograft models (23). Tumor size was measured weekly until the sacrifice of the animals. One week after the third administration (day 21), a significant delay in tumor growth was observed in CHC001PDX mice treated with ET-743 (p = 0.04, Figure 2A). Similar results were obtained in EGI-1 xenografts (p = 0.001) (Figure 2B). Tumors harvested from CHC001PDX and EGI-1 xenografts treated with either PBS or ET-743 were subjected to immunohistochemical analysis for Ki67/MIB1 proliferation index detection. A reduction in the number of proliferating cells in treated compared to untreated CHC001PDX mice was revealed (Figure 2C; Ki67: p = 0.05). In EGI-1 xenografts, we found a moderate inhibition of proliferation in treated compared to untreated mice (Figure 2D). By contrast, we found a weakly but not significant increase of apoptotic cells *in vivo* (data not shown).

**Figure 3:**
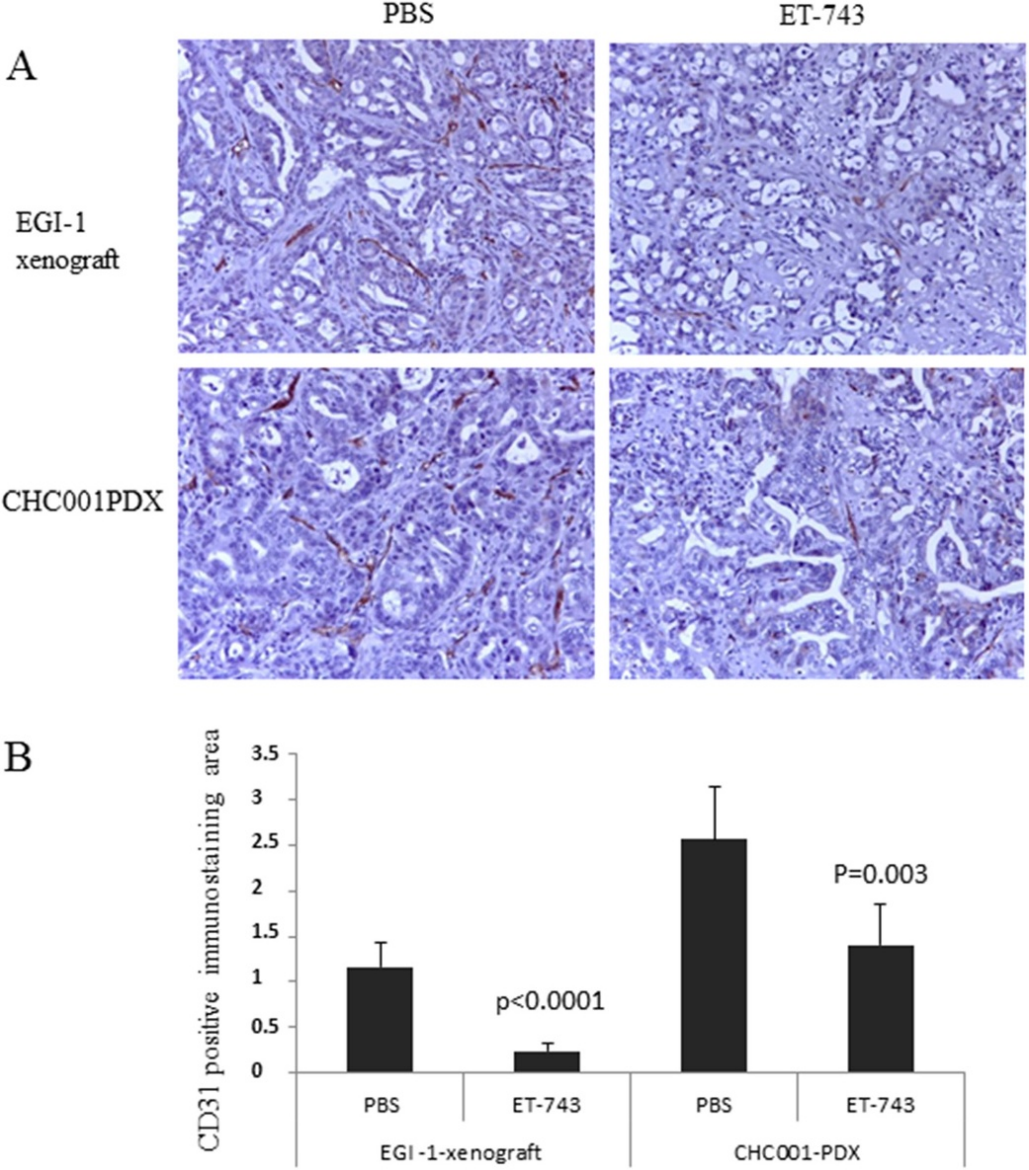
**Representative CD31 expression (A) and quantification (B) on EGI-1 xenografts and CHC001PDX upon ET-743 treatment. A**. Sections derived from treated and untreated (PBS) EGI-1 xenografts and CHC001PDX were subjected to immunohistochemistry and stained with anti-mouse CD31. **B**. CD31 quantification was performed on 10 z-stack images for each slide at 20x magnification by calculating the positively stained vessel area.

We observed that ET-743 treated-tumors were macroscopically less vascularized compared with the control cohorts of mice. Thus, we verified the status of tumor vasculature at the microscopic level. Tumor sections from control and ET-743 treated CHC001PDX/EGI-1 xenografts were stained for the endothelial marker CD31. A significant reduction in the number of tumor blood vessels was observed in ET-743-treated mice (Figure 3) (p < 0.0001 and p = 0.003 for EGI-1-xenograft and CHC001PDX respectively). Further, we evaluated the *in vitro* effect of drug on human endothelial Huvec cells after 72 hours of treatment with ET-743. A potent inhibitory effect on proliferation was revealed, with an IC50 of 0.16 nM, comparable to those found in the most sensitive BTC cells (data not shown).

### ET-743 modulates genes involved in cell adhesion processes, in stress response, and pathways involved in cholangiocarcinogenesis

**Figure 4:**
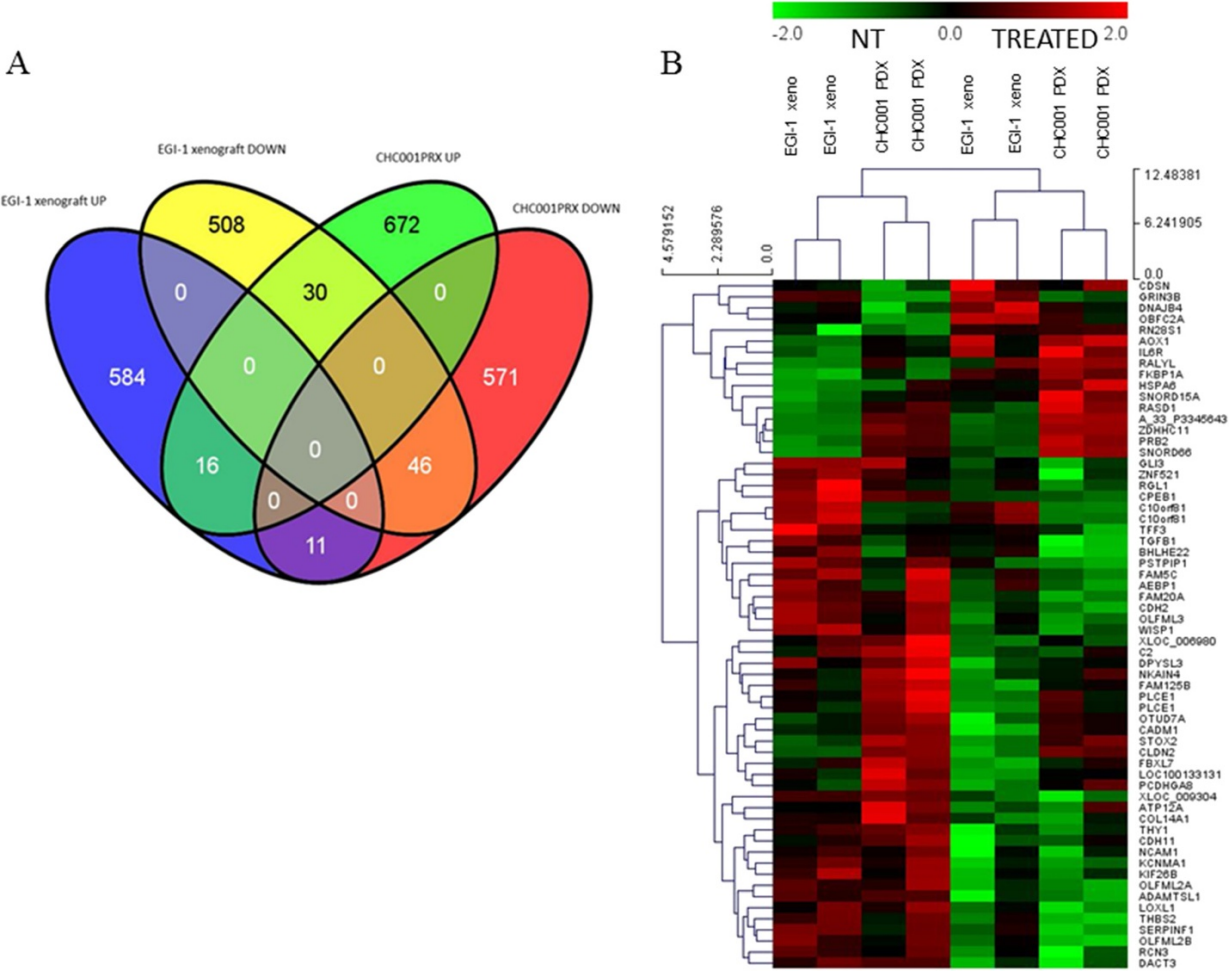
**Gene expression analysis of the** ***in vivo*** **models after treatment with ET-743. A**. Venn Diagramm showed the differentially expressed probes distribution in the two *in vivo* models. **B**. Hierarchical clustering applied to the expression matrix of genes modulated in a concordant manner by the treatment in both CHC001PDX/EGI-1 models, using Euclidean distance as similarity metrics and average linkage as linkage method. Genes were median center and divided by standard deviation. NT: not treated mice; TREATED: treated with ET-743.

The impact of ET-743 on gene expression was assessed in *in vivo* models. Upon ET-743 treatment, 1346 differentially expressed probes were identified in CHC001PDX, 628 down-regulated and 718 up-regulated. In EGI-1 xenograft, 1195 differentially expressed probes were identified, 584 down-regulated and 611 up-regulated. Gene Ontology analysis was performed on the two datasets (significant p-value <0.01), considering up and down-regulated genes separately. We identified at least 20 deregulated biological processes upon ET-743 treatment in CHC001PDX and EGI-1 xenografts (Additional file 5: Table S2). Comparing the two models, we found an overlap of differentially expressed probes upon ET-743 treatment, whose expression is able to subdivide treated from untreated samples by an unsupervised hierarchical clustering analysis (Figure 4). In particular there are 46 common down-regulated genes, 12 out of them (i.e., CADM1, WISP1, CDH2, COL14A1, THY1, CDH11, PSTPIP1, PCDHGA8, THBS2, CLDN2, NCAM1, AEBP1) are involved in cell adhesion processes; further, CDH2/N-Cadherin, a member of the trefoil factor (*TFF*) gene family, the TFF3 gene, transforming growth factor-β1 (TGF-β1) and N-CAM1, have been previously associated to BTC [29–34]. We also found 16 common up-regulated genes, 5 out of them (HSPA6, OBFC2A, DNAJB4, IL6R, AOX1) are involved in response to stress (Additional file 6: Table S3). Results obtained by microarray experiments were validated by quantitative RT-PCR for some selected deregulated genes (CDH11, TFF3, C FAM5C, IL6R, KCMNA1) (data not shown). Further, pathway analysis of both datasets was performed using PathwayMiner software (http://www.biorag.org); using three different databases, we found 12 common pathways significantly deregulated by ET-743 (Additional file 7: Table S4): among them the IL-6, the Sonic Hedgehog and the Wnt signaling pathways are involved in cholangiocarcinogenesis [35–38].

## Discussion

ET-743 has been approved for treatment of ovarian cancer and soft-tissue sarcoma with significant activity in liposarcomas and leiomyosarcomas, both as a single agent and in combination with other drugs. Here, we demonstrated that ET-743 has an antitumor activity both *in vitro* and *in vivo* in preclinical models of human BTC. We reported that ET-743 inhibits BTC *in vitro* cell growth. Interestingly, the most responsive models to ET-743 were represented by the HuH28 cell line, previously demonstrated to be resistant to GEM *in vitro*[28] and the ICP-2 primary tumor cell cultures derived from GEMOX-resistant patient. Cell cycle studies on ET-743-treated BTC cells, demonstrated that the chemotherapy acts by modifying the cell cycle status, mostly inducing an increase of sub-G_0_ phase cell fraction and triggering apoptosis as preferential mechanism. It has been demonstrated that ET-743 causes double-strand DNA damage, detectable by Ser139 phosphorylation of the histone H2A.x. This event triggers a cascade leading to the activation of ATM, and consequently of p53 [39, 40]. Although BTC cell lines display different p53 mutational status, and in particular TFK-1 cells are p53-deleted, we did not find any significant correlation between response to ET-743 and p53 mutational status in these cell lines. This is in agreement with a previous report [7].

In recent years, clinical validation of anticancer therapies has benefited from *in vivo* models derived from the direct implantation of human tumors in immunocompromized mice. These preclinical models present the advantage to recapitulate the biological characteristics of the primary tumor and represent a useful tool for the study the biology of tumors and the clinical response to new therapeutic approaches [13, 41, 42]. To investigate the *in vivo* antitumor activity of ET-743, we created BTC mouse xenograft models. By using one of such PDX models and a conventional xenograft from a BTC cell line (EGI-1), we showed that three administrations of ET-743 are sufficient to cause a significant delay of tumor growth compared to the respective untreated control groups. Tumor growth delay by ET-743 could be ascribed to a decrease in the number of tumor proliferating cells, and a net decrease of tumor vessel formation. We were not be able to demonstrate induction of apoptosis *in vivo* suggesting that *in vitro* the activity could be more directed to BTC cells, but *in vivo* ET-743 is also directed against the tumor microenvironment, in particular by decreasing the tumor-associated macrophages and down-regulation of cytokines, chemokines and angiogenic factors [16].

The transcriptional signature upon ET-743 treatment revealed a panel of common deregulated genes. Among them, 12 cell adhesion related genes were down-regulated, while 5 stress response genes were up-regulated. Interestingly, ET-743 reduced the expression of WISP1, which has been associated to a more aggressive phenotype of BTC [43]; another down-regulated gene, TFF3, a member of the trefoil factor (*TFF*) gene family, was demonstrated to be associated with tumor progression in colorectal cancer [30] and in BTC [44]. ET-743 also induced the down-modulation of TGF-β1, highly expressed in BTC and contributing to the angiogenic switch in preclinical models [32]. Moreover, Shimizu and coll. [31] provided evidence that TGF- β1 supports BTC cell growth, indicating its potential role as a molecular target. Further, we found a deregulation of genes involved in the IL-6, the Sonic Hedgehog and the Wnt signaling pathways, all demonstrated to be involved in cholangiocarcinogenesis [35–38].

As ET-743 inhibited the neoplastic compartment but also affected the tumor micro-environment, it is likely to be more effective for those malignancies as BTC in which chronic inflammation is known to contribute in tumor progression by causing myelomonocitic infiltration and producing chemokines and cytokines. However, these aspects are more difficult to be reproduced in preclinical models and need to be explored in a clinical setting.

## Conclusions

In conclusion, our data suggest that ET-743 could be a promising alternative chemotherapy for BTC treatment, providing a strong rationale in the design of a clinical trial to evaluate the activity of ET-743 in BTC patients resistant to GEM-based therapeutic regimens.

## Electronic supplementary material

Additional file 1: Table S1: Comparison of IC50 values after treatment with GEM or ET-743 on BTC cells. (DOCX 49 KB)

Additional file 2: Figure S1: Representative histograms of distributions of TFK-1, WITT, and KMCH on cell cycle phases. NT: not treated cells. (TIFF 59 KB)

Additional file 3: Figure S2: Representative Dot plots analysis of apoptosis induction in TGBC1, TFK-1, WITT, and KMCH cell lines.NT, not treated; ET-743, treated with drug. (TIFF 132 KB)

Additional file 4: Figure S3: ET-743 induces the phosphorylation of proteins involved in DNA damage-repair in BTC cell lines. Cells were treated with 5 nM of ET-743 for 24 hours followed by Western blot analysis to investigate the phosphorylation status of ATM, p53, and H2A.x. (TIFF 136 KB)

Additional file 5: Table S2: Gene Ontology of ET-743 treated vs untreated in vivo models. **A**. CHC001PDX treated vs CHC001PDX untreated: BPall pv < 0.01 - BG Agilent HumanGenome. **B**. EGI-1 xenograft-treated vs EGI1 xenograft-untreated: BPall pv < 0.01 - BG Agilent HumanGenome. (XLSX 14 KB)

Additional file 6: Table S3: Common deregulated (46 down-regulated and 12 up-regulated) elements between CHC001PDX and EGI-1 xenograft. (XLSX 22 KB)

Additional file 7: Table S4: Common deregulated pathways obtained by PathwayMiner software, using three different databases. A p value <0.05 was considered as statistically significant. (DOCX 15 KB)

## Abbreviations

ET-743: Ecteinascidia turbinate-743
BTC: Biliary tract carcinoma
IL-6: Interleukin-6
GEM: Gemcitabine
OS: Overall survival
VEGF: Vascular endothelial growth factor
TAMs: Tumor-associated macrophages
ECC: Extrahepatic cholangiocarcinoma
ICC: Intrahepatic cholangiocarcinoma
GBC: Gallbladder carcinoma
FBS: Fetal bovine serum
DMSO: Dimethyl sulfoxide
PDX: Patient derived xenograft
PR: Primitive
NOD/Shi-SCID: Non-Obese Diabetic/severe combined immunodeficient
PBS: Phosphate buffered saline
PI: Propidium Iodide
APC: Allophycocyanin
HRP: Horseradish peroxidase
GEP: Gene expression profiling
SEM: Standard error of the mean.
