# Supplementary material for: Anti-cancer effect and gene modulation of ET-743 in human biliary tract carcinoma preclinical models
Source: BMC Cancer. 2014 Dec 5;14:918. doi: 10.1186/1471-2407-14-918 (PMC4289395; doi:10.1186/1471-2407-14-918)
Supplement: Supplementary file 1 — Additional file 1: Table S1: Comparison of IC50 values after treatment with GEM or ET-743 on BTC cells. (DOCX 49 KB) [file 12885_2014_5134_MOESM1_ESM.docx]

**Supplementary table 1: Comparison of IC50 values after treatment with GEM or ET-743 on BTC cells**

**
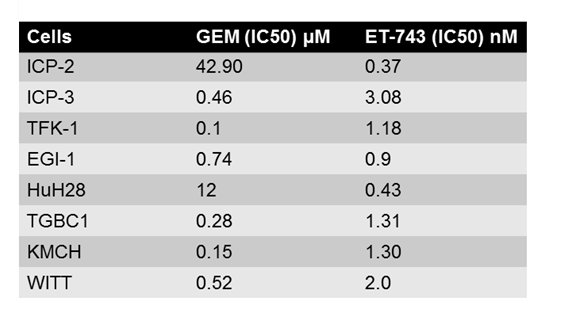
**

ICP-2/3 primary cells derived from 3 intrahepatic cholangiocarcinoma patients. TFK-1, WITT and EGI-1: extrahepatic cholangiocarcinoma cell lines; HUH28 intrahepatic cholangiocarcinoma cell line; TGBC1: gallbladder carcinoma cell line; KMCH intrahepatic mixed with hepatocarcinoma cell line.
