# Supplementary figures and images for: Anti-cancer effect and gene modulation of ET-743 in human biliary tract carcinoma preclinical models
Source: BMC Cancer. 2014 Dec 5;14:918. doi: 10.1186/1471-2407-14-918 (PMC4289395; doi:10.1186/1471-2407-14-918)

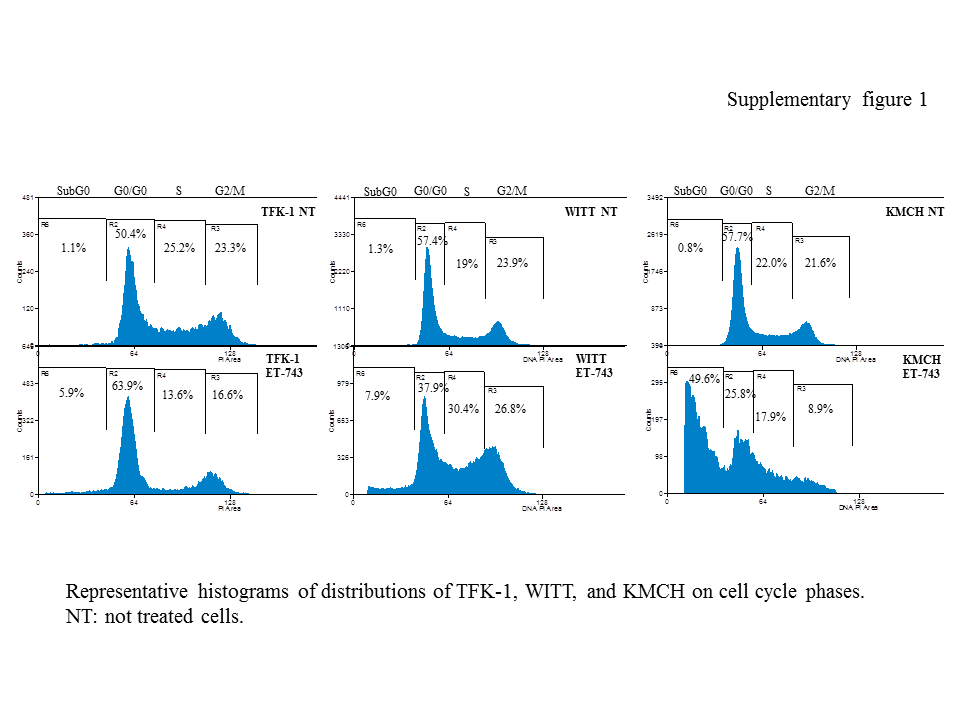

Supplement: Supplementary file 2 — Additional file 2: Figure S1: Representative histograms of distributions of TFK-1, WITT, and KMCH on cell cycle phases. NT: not treated cells. (TIFF 59 KB) [file 12885_2014_5134_MOESM2_ESM.tiff]

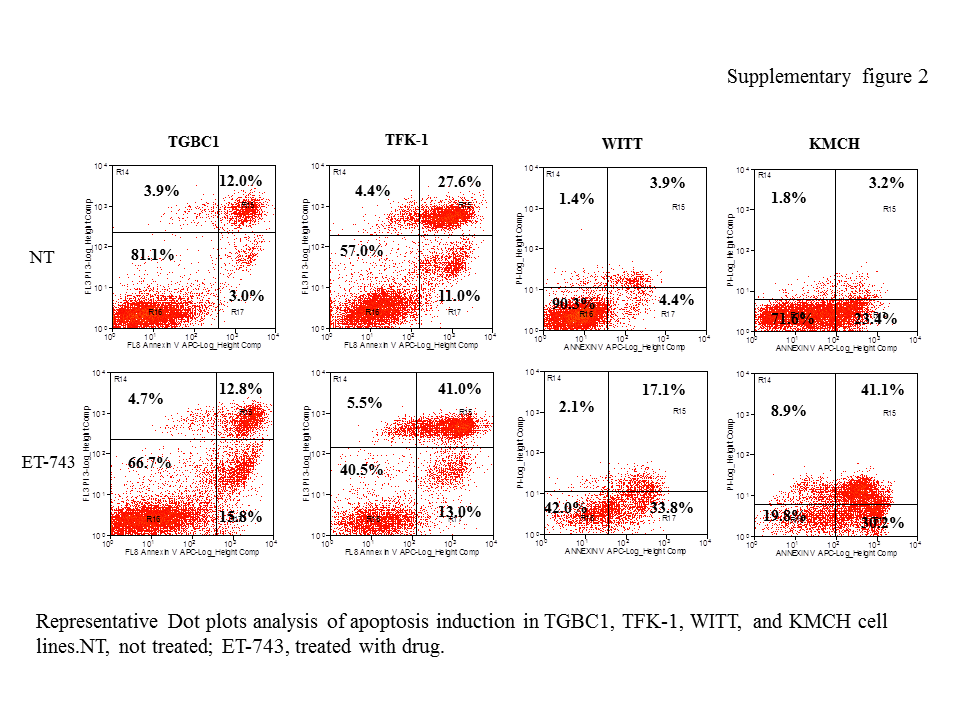

Supplement: Supplementary file 3 — Additional file 3: Figure S2: Representative Dot plots analysis of apoptosis induction in TGBC1, TFK-1, WITT, and KMCH cell lines.NT, not treated; ET-743, treated with drug. (TIFF 132 KB) [file 12885_2014_5134_MOESM3_ESM.tiff]

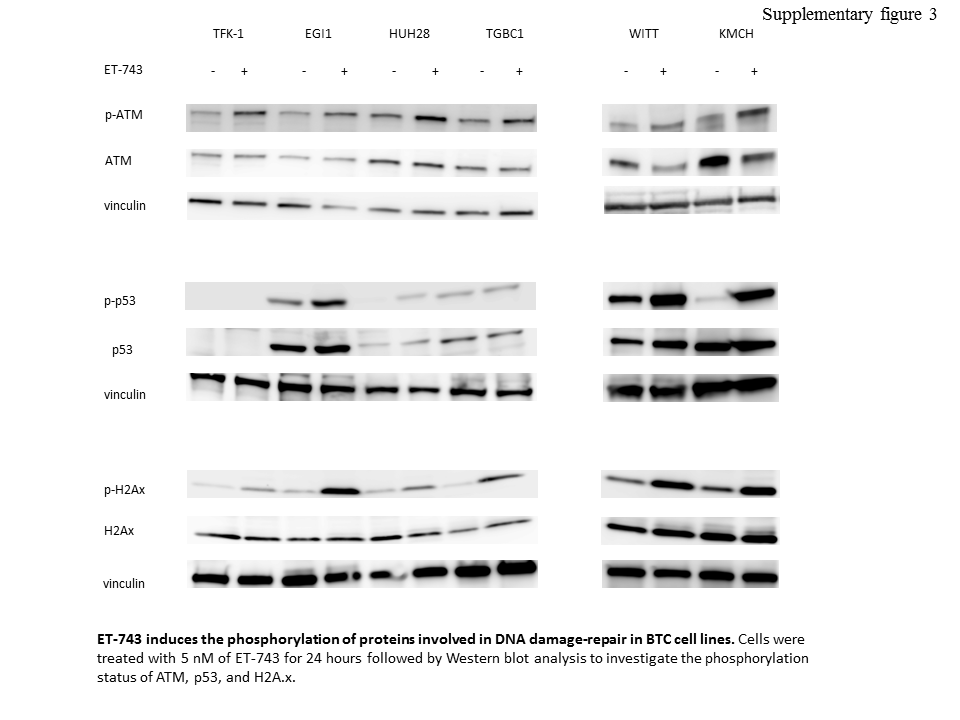

Supplement: Supplementary file 4 — Additional file 4: Figure S3: ET-743 induces the phosphorylation of proteins involved in DNA damage-repair in BTC cell lines. Cells were treated with 5 nM of ET-743 for 24 hours followed by Western blot analysis to investigate the phosphorylation status of ATM, p53, and H2A.x. (TIFF 136 KB) [file 12885_2014_5134_MOESM4_ESM.tiff]
