# Supplementary material for: Anti-cancer effect and gene modulation of ET-743 in human biliary tract carcinoma preclinical models
Source: BMC Cancer. 2014 Dec 5;14:918. doi: 10.1186/1471-2407-14-918 (PMC4289395; doi:10.1186/1471-2407-14-918)
Supplement: Supplementary file 7 — Additional file 7: Table S4: Common deregulated pathways obtained by PathwayMiner software, using three different databases. A p value <0.05 was considered as statistically significant. (DOCX 15 KB) [file 12885_2014_5134_MOESM7_ESM.docx]

**Supplementary table 4.** Common deregulated pathways obtained by PathwayMiner software, using three different databases. A p value <0.05 was considered as statistically significant.

| **Source** | **Pathway Name** | **p-value CHC001PDX**  **(<0.05)** | **p-value EGI-1 xenograft**  **(<0.05)** |
| --- | --- | --- | --- |
| **BIOCARTA** | FOSB gene expression and drug abuse | 0.02372 | 0.02842 |
|  | IL 6 signaling pathway | 0.0469 | 0.02842 |
|  | Activation of Csk by cAMP-dependent Protein Kinase Inhibits Signaling through the T Cell Receptor | 0.02372 | 0.02842 |
|  | Sonic Hedgehog (Shh) Pathway | 0.00157 | 0.02842 |
| **GENMAPP** | Hs Wnt signaling | 0.01913 | 0.00007 |
|  | Hs GPCRDB Class A Rhodopsin-like | 0.03188 | 0.00573 |
|  | Hs Smooth muscle contraction | 0.00031 | 0.01638 |
|  | Hs G Protein Signaling | 0.0224 | 0.04094 |
|  | Hs G1 to S cell cycle Reactome | 0.0224 | 0.04094 |
| **KEGG** | Cell adhesion molecules (CAMs) | 0.000001 | 0.00505 |
|  | Cytokine-cytokine receptor interaction | 0.01344 | 0.00005 |
|  | Cell Communication | 0.00001 | 0.00447 |
